# 9.G. Workshop: Health in all Policies: key driver for better health still awaiting of greater governing stewardship

**DOI:** 10.1093/eurpub/ckac129.575

**Published:** 2022-10-25

**Authors:** Organised by: EUPHA-HIA, EUPHA-PHPP, EUPHA-ECO, EUPHA-LAW, Chair persons: Piedad Martin-Olmedo (EUPHA-HIA), Monica O'Mullane (Ireland)

## Abstract

Healthy public policies are those that take accountability of all possible health impacts, acknowledging the causal pathways resulting from the modification of upstream health determinants (e.g. transport strategies, etc.), and related risk factors downstream (e.g. air pollutants). The strategy of Health in All Policies (HiAP), promoted by the World Health Organization (WHO) and adopted by the European Union (EU) in 2006, reinforced the need to reduce inequalities and improve health and wellbeing as essential pillars for a sustainable economic development. Central to HiAP is the notion that health is not only the responsibility of the health sector, but also a shared responsibility with many other sectors. In this context, Health impact Assessment (HIA) was proposed as the combination of methods to support HiAP implementation by providing scientific evidence on the positive and negative effects that any new proposal may have on health and health equity. The COVID-19 pandemic, and the climate change threat, are two of the main challenges that emphasise the need of integrated responses across many sectors to mitigate not only effects on health and inequalities, but also in the economy. However, HiAP and HIA implementation remains almost at a conceptual level, with a few remarkable exceptions in Europe. One of the most relevant reported barriers contributing to this uneven HiAP implementation is the lack of political stewardship and commitment. The difficulties in applying the guiding principles of HiAP (and consequently of HIA) at local, regional, or national governance level are in many cases linked to a conflict between the right to work and mobilization of the economy, with the right to health and reduction of inequities. This is where the role and drive of public health actors comes across, as HiAP requires public health professionals to build partnerships and engage meaningfully with the sectors affecting the social determinants of health and health equity, external to the health sector. A good proxy example to HiAP implementation, facilitating local and regional initiatives with communities, is the WHO initiative of Healthy Cities. The present panel discussion intends to analyse, from different perspectives, why HiAP has not gained a meaningful place within governing contexts, the current and future status of the intersectoral approach, and the advocacy role of public health in this context. The session is scheduled with a first overview presentation followed by a debate framed around the following aspects:

– Different perceptions regarding the concrete implementation of HiAP at all political levels.

– Perceived barriers or trade-offs for a broader implementation of HiAP.

– Role of public health actors in the implementation of HiAP at a strategic, policy level, and how it could gain a more prominent role.

– Level of understanding and awareness of the utility of HIA for HiAP implementation by public health actors.

**Key messages:**

• HiAP, a recognised approach requiring all sectors to address decisions’ health and equity implications for reaching better global health, has not yet been implemented with an overarching vision.

• Public Health actors can disentangle political and technical aspects, seeking for synergisms, and clarifying to non-health sectors the complexity and interrelatedness of social health determinants.

